# Chemokine C-C Motif Ligand 7 (CCL7), a Biomarker of Atherosclerosis, Is Associated with the Severity of Alopecia Areata: A Preliminary Study

**DOI:** 10.3390/jcm10225418

**Published:** 2021-11-19

**Authors:** Anna Waśkiel-Burnat, Anna Niemczyk, Leszek Blicharz, Paulina Chmielińska, Michał Zaremba, Aleksandra Gąsecka, Krzysztof J. Filipiak, Małgorzata Olszewska, Lidia Rudnicka

**Affiliations:** 1 Department of Dermatology, Medical University of Warsaw, 02-008 Warsaw, Poland; anna.waskiel@wum.edu.pl (A.W.-B.); anna.stochmal@wum.edu.pl (A.N.); leszek.blicharz@wum.edu.pl (L.B.); paulawierzb@gmail.com (P.C.); zaremba_michal@wp.pl (M.Z.); malgorzata.olszewska@wum.edu.pl (M.O.); 2 1st Chair and Department of Cardiology, Medical University of Warsaw, Banacha 1a, 02-097 Warsaw, Poland; aleksandra.gasecka@wum.edu.pl; 3 Institute of Clinical Sciences, Maria Sklodowska-Curie Medical Academy, Pałac Lubomirskich, Plac Żelaznej Bramy 10, 00-136 Warsaw, Poland; krzysztof.filipiak@uczelniamedyczna.com.pl

**Keywords:** cardiovascular disease, coronary artery disease, heart disease, hair loss, stroke, myocardial infarction, peripheral arterial disease

## Abstract

Alopecia areata is an autoimmune, inflammatory form of non-scarring hair loss that may affect any hair-bearing area. Recently, an increased risk of cardiovascular disorders has been described in patients with alopecia areata. The aim of the study was to evaluate the serum concentrations of proinflammatory proteins associated with atherosclerosis (chemokine C-C motif ligand 4; CCL4, chemokine C-C motif ligand 7, CCL7; and sortilin, SORT1), and cardiovascular risk (myeloperoxidase, MPO; interleukin 1 receptor-like 1, IL1RL1; and growth differentiation factor 15, GDF15) in patients with alopecia areata without symptoms or prior cardiovascular disease in comparison with healthy controls. Sixty otherwise healthy patients with alopecia areata and twenty control subjects matched for age, gender, and body mass index (BMI) were enrolled in the study. No significant differences in the serum levels of MPO, IL1RL1, CCL4, CCL7, SORT1, and GDF15 were detected between patients with alopecia areata and healthy controls. A positive correlation was found between the serum concentration of CCL7 and the severity of alopecia areata (r = 0.281, *p* = 0.03), while GDF15 correlated with age at the disease onset (r = 0.509, *p* < 0.0001). The results of the present study suggest that the severity of alopecia areata may be associated with an increased risk of atherosclerosis.

## 1. Introduction

Numerous systemic conditions are associated with future cardiovascular events. Well-established risk factors for the development of cardiovascular diseases include diabetes, hypertension, dyslipidemia, and a positive family history as well as non-independent risk variables, such as smoking and being overweight. Chronic inflammation was also postulated to play an important role in the development and propagation of cardiovascular conditions [1].

Alopecia areata is an autoimmune type of non-scarring hair loss [2]. The prevalence of the disease in the general population is 0.2% with a lifetime risk being approximately 1.7–2.1% [3]. Alopecia areata is caused by the infiltrates of T helper cells, cytolytic T cells, plasmacytoid dendritic cells, and natural killer cells around the lower part of the hair bulb in the anagen phase, which induce the collapse of the hair follicle immune privilege and subsequent hair loss [4]. 

Alopecia areata has been considered as an organ-specific disorder limited to the hair follicles. However, numerous studies indicate that alopecia areata is associated with a systemic immune activation and the dysregulation of multiple proinflammatory cytokines, including Interleukin 1 beta (IL-1β), IL-6, tumor necrosis factor alpha (TNF-α), and interferon gamma (IFN-γ) [5]. It has also been suggested that chronic inflammation in alopecia areata may result in an increased risk of developing cardiovascular comorbidities, such as coronary artery disease and stroke [5,6].

Numerous novel proinflammatory proteins are associated with atherosclerosis and cardiovascular risk, including chemokine C-C motif ligand 4 (CCL4), chemokine C-C motif ligand 7 (CCL7), sortilin (SORT1), myeloperoxidase (MPO), interleukin 1 receptor-like 1 (IL1RL1), and growth differentiation factor 15 (GDF15) [7]. An increased serum level of these markers was reported in patients with atopic dermatitis [7]. Some common pathogenic mechanisms underlying alopecia areata and atopic dermatitis, including the role of IL-4, IL-13, and filaggrin [8], may suggest a similar cardiovascular pattern in the disorders.

The aim of the study was to evaluate the serum concentrations of proinflammatory proteins associated with atherosclerosis (CCL4, CCL7, and SORT1) and cardiovascular risk (MPO, IL1RL1, and GDF15) in patients with alopecia areata without symptoms or prior cardiovascular disease in comparison with healthy controls.

## 2. Materials and Methods

### 2.1. Patients

Patients diagnosed with alopecia areata (41 women and 19 men) who consulted in our department between March 2021 and May 2021 were screened for inclusion in this study. The following inclusion criteria were determined: patients aged 18 years and above diagnosed with alopecia areata. Exclusion criteria were: symptoms of cardiovascular disease, autoimmune diseases other than alopecia areata, malignancy, pregnancy, breastfeeding, chronic coronary artery disease, a history of acute coronary syndrome or coronary revascularization, heart failure with reduced ejection fraction, a cardiac condition requiring surgery, a history of stroke, a transient ischemic attack or vascular revascularization, severe grade II or III arterial hypertension, diabetes, and dyslipidemia. The control group comprised healthy individuals matched for age, gender, and body mass index (BMI) with the same exclusion criteria.

Demographic data and clinical variables, such as age, gender, smoking, a family history of cardiovascular disease, weight, and height, were collected in all individuals. The BMI was calculated as weight (kg)/height^2^ (m) [9]. Additionally, data concerning the age at which the first episode of hair loss occurred, the number and duration of the present episode of hair loss were recorded in patients with alopecia areata. The severity of hair loss was assessed with the severity of an alopecia tool (SALT) [10]. Subsequently, the patients were divided into two groups: (1) with SALT score < 50% and (2) with SALT score ≥ 50%. The activity of hair loss was evaluated and defined as: (1) progressive alopecia areata, an increase in total hair loss of more than 5%; (2) stable, a change in total hair loss of less than 5%; and (3) remitting alopecia areata, a decrease in total hair loss of more than 5% over the month prior to the examination [11].

The serum levels of total cholesterol, low-density lipoprotein cholesterol (LDL cholesterol) cholesterol, high-density lipoprotein cholesterol (HDL cholesterol), triglycerides, and fasting glucose were measured in both groups.

### 2.2. Measurement of Atherosclerosis and Cardiovascular Risk Markers

Venous blood was collected using a 19G needle and centrifuged to obtain the serum, which was stored at −80 °C. The serum concentrations of MPO, IL1RL1, CCL4, CCL7, SORT1, and GDF15 were measured using a commercially available ELISA kit (EIAab, Wuhan, China), according to the manufacturer’s instructions.

### 2.3. Statistical Analysis

The Shapiro–Wilk test was used to evaluate data for the normality of distribution. Normally distributed variables were presented as the mean ± standard deviation (SD). Non-normally distributed variables were shown as the median and interquartile range (IQR). Categorical data were compared with the chi-squared test and presented as a number of cases and percentages. The Student’s t-test and the Mann–Whitney U test were used to analyze parametric and nonparametric continuous variables, respectively. The correlation coefficient Spearman’s rank test was used to assess possible linear associations between two continuous variables. To compare the variables according to the disease activity, one-way ANOVA was used to analyze normally distributed variables and the Kruskal–Wallis test was used for variables that were not normally distributed. The values of *p* < 0.05 were considered statistically significant.

All statistical analyses were performed with STATISTICA 13.1 (StatSoft, Cracow, Poland).

### 2.4. Ethics Committee Approval

The study protocol conformed to the principles of the World Medical Association’s Declaration of Helsinki and was approved by the Medical University of Warsaw Review Board for Ethics in Human Research (KB/142/2020). Written informed consent was obtained from all participants.

## 3. Results

Sixty patients with alopecia areata and twenty control subjects were enrolled in the study. The groups did not differ with respect to age, sex distribution, and BMI. No differences were revealed as regards smoking, a family history of cardiovascular diseases, lipid profile, and fasting glucose. No significant differences in the serum concentrations of MPO, IL1RL1, CCL4, CCL7, SORT1, and GDF15 were detected between patients with alopecia areata and healthy controls (Table 1).

**Figure 1:**
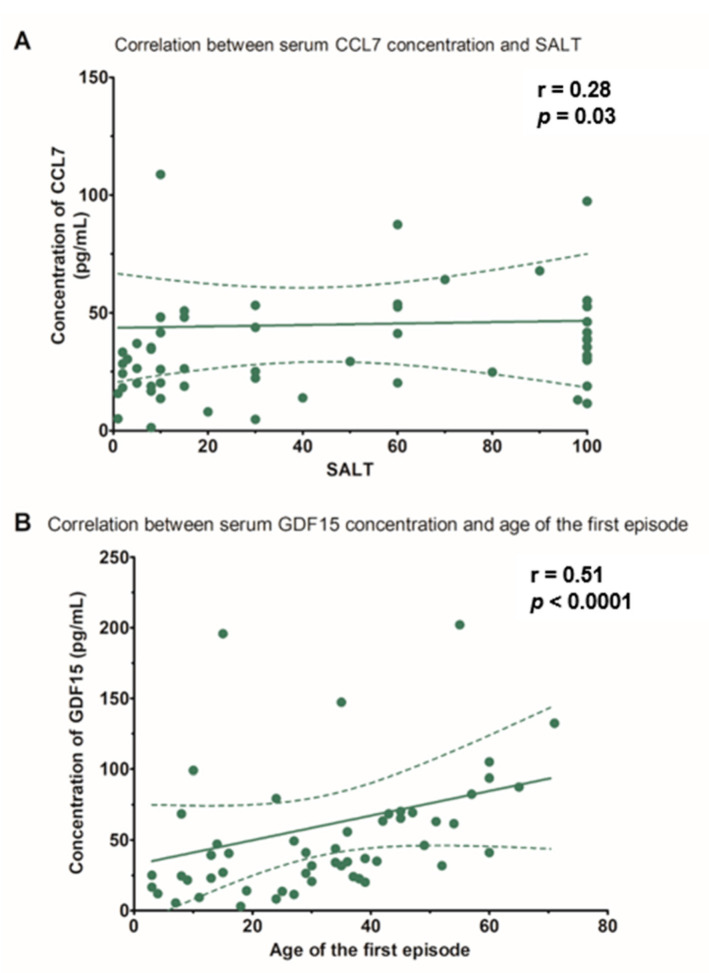
(**A**) Correlation between the serum concentrations of chemokine C-C motif ligand 7 (CCL7) and the severity of hair loss in patients with alopecia areata, evaluated with the severity of alopecia tool (SALT). (**B**) Correlation between the serum concentrations of growth differentiation factor 15 (GDF15) and patient age at the first episode of alopecia areata.

However, a significant positive correlation was found between CCL7 and SALT score (r = 0.281, *p* = 0.03) (Table 2 and Figure 1A). In addition, the GDF15 serum level correlated with the age at the disease onset (r = 0.509, *p* < 0.0001) (Table 2 and Figure 1B).

The comparison between patients with a SALT score < 50% and healthy subjects revealed no significant differences in the serum levels of various atherosclerosis and cardiovascular markers (Table 3).

Moreover, no significant differences were observed between patients with a SALT score ≥ 50% and the healthy controls (Table 4).

No significant differences in the serum levels of MPO, IL1RL1, CCL4, CCL7, SORT1, and GDF15 were present between patients with active, stable, and remitting alopecia areata. However, a decreased serum level of HDL cholesterol was identified in patients with active and stable alopecia areata compared to individuals with remitting hair loss (Table 5).

## 4. Discussion

Alopecia areata is an autoimmune inflammatory condition. Thus, it was hypothesized that it might be associated with an increased risk of cardiovascular disorders [6]. However, data concerning this association are inconsistent. A cross-sectional study conducted by Conic et al. [6], which comprised 33,130 patients with alopecia areata and 5,246,350 non-alopecia areata control subjects, revealed that coronary artery disease (5.5% vs. 1.8%) and stroke (0.45% vs. 0.31%) were more commonly observed in patients with alopecia areata in comparison with the controls. No significant difference occurred in the prevalence of myocardial infarction between both groups (2.2% vs. 2.1%). Jun-Won et al. [12] evaluated the risk of myocardial infarction in patients with alopecia areata during a 12-year follow up. During the early phase of observation, the cumulative incidence of acute myocardial infarction in individuals with alopecia areata was lower compared to the control subjects (incidence rate ratio, 0.52 (95% CI, 0.42–0.65) between 2 and 4 years). However, in the later phase of the follow up period, it increased and was greater than in the controls (incidence rate ratio, 2.06 (95% CI, 1.71–2.45) between 8 and 10 years). A study performed by Kang et al. [13] revealed higher incidence rates of stroke during a 3-year follow up period in patients with alopecia areata compared to controls (5.44 vs. 2.75 per 1000 person per year). On the contrary, a 10-year retrospective cohort study by Lee et al. [14], which included 3770 patients with alopecia areata and 18,850 controls, revealed no significant differences in the incidence of various cardiovascular diseases between those two groups. Heart failure was reported in 0.3% of patients with alopecia areata and in 0.2% of the healthy controls. Angina pectoris, acute myocardial infarction, and chronic myocardial infarction were observed in 1.6%, 0.1%, and 0.4% of patients with alopecia areata, respectively. As regards control subjects, they were present in 1.4%, 0.2%, and 0.4%, respectively. Moreover, peripheral vascular disease and atherosclerosis were found in 0.9% of patients with alopecia areata and 1.1% of control subjects, while stroke was reported in 2.3% of patients with alopecia areata and in 2.1% of controls. The analysis of 1377 patients with alopecia areata and 4131 matched controls conducted by Huang et al. [15] revealed a lower frequency of ischemic stroke (0.7% vs. 1.3%) and myocardial infarction (2.0% vs. 2.2%) in patients with alopecia areata than in the controls.

Several biomarkers have been identified as useful in the diagnosis, management, and prognostic stratification of patients with cardiovascular diseases. Only a limited number of studies have been published as regards cardiovascular biomarkers in patients with alopecia areata to date. Troponin I is an important marker of cardiomyocyte injury. However, it may be also elevated in individuals with subclinical cardiac involvement. Wang et al. [16] reported higher plasma cardiac troponin I levels in patients with alopecia areata in comparison with patients with androgenetic alopecia and healthy controls. Moreover, Glickman et al. [7] demonstrated that the serum levels of various atherosclerosis and cardiovascular biomarkers, including E193 selectin, matrix metalloproteinases, lectin-type oxidized LDL 194 receptor 1, myeloperoxidase, fatty acid binding protein, P195 selectin, oncostatin M, proteinase-3, peptidoglycan recognition protein 1, and caspase-3, were increased in patients with moderate-to-severe alopecia areata in comparison with the controls. Moreover, the authors reported a positive correlation between various markers and disease severity. In the present study, no significant differences in the serum levels of various atherosclerosis and cardiovascular markers (MPO, IL1RL1, GDF15, CCL4, CCL7, and SORT1) were observed between patients with alopecia areata and healthy controls. Such inconsistency among studies may be associated with different cardiovascular risk profiles of the individuals included into the analyses. Numerous systemic conditions, such as dyslipidemia or diabetes, as well as smoking and a family history are associated with the increased risk of cardiovascular disease development. Thus, their presence must be taken into consideration when evaluating the independent impact of alopecia areata on cardiovascular risk.

CCL7, also known as monocyte chemotactic protein-3, belongs to the monocyte chemotactic protein subfamily of C-C chemokines. It is a potent chemoattractant for a variety of leukocytes, including monocytes, eosinophils, basophils, dendritic cells, natural killer cells, and activated T lymphocytes [17]. The role of CCL7 in vascular pathologies, including atherosclerosis, has been reported. The expression of CCL7 in human atherosclerotic plaques is induced by oxidized LDL [18]. Moreover, it was described that CCL7 induced coronary smooth muscle cell proliferation [18], which was observed during early atherogenesis [19]. Increased serum levels of CCL7 also constitute a predictor of increased cardiovascular morbidity and mortality in patients with acute myocardial infarction [20]. In the present study, there was no significant difference in the serum levels of CCL7 between otherwise healthy patients with alopecia areata and healthy controls. However, in patients with alopecia areata, they correlated with disease severity. The lack of a significant difference in the serum levels of CCL7 between patients with alopecia areata and healthy controls may be associated with the low number of patients included into the analysis.

GDF15 belongs to the transforming growth factor-β (TGF-β) cytokine superfamily, which has been shown to regulate the inflammatory and angiogenesis pathways. GDF-15 exhibits various and even contradictory functions under various circumstances, including proapoptotic/antiapoptotic, proangiogenetic/antiangiogenetic, and proinflammatory/anti-inflammatory properties [21]. It has been suggested that GDF-15 plays an important role in the pathogenesis of atherosclerosis. The concentration of GDF15 was shown to correlate with the severity of coronary artery disease in patients with a suspected myocardial infarction and was an independent predictor of future cardiovascular events in those patients [22]. In our study, the serum concentration of GDF15 correlated with the age at the onset of alopecia areata. Since age is a well-established classic risk factor of cardiovascular disease, it could be hypothesized that higher concentrations of GDF15 along with age contribute to the development of atherosclerosis in patients with alopecia areata.

The primary limitation of our study is a relatively small sample size, leading to the lack of statistical power to show the differences in the serum concentrations of proinflammatory markers between patients with alopecia areata and the controls. For example, patients with alopecia areata were characterized by nominally higher concentrations of CCL4, compared to the controls, which might become significant in a larger cohort. Finally, the study did not show a direct association between the serum concentrations of proinflammatory biomarkers and cardiovascular events in patients with alopecia areata, which would require a long-term observation period (e.g., 10 years). Altogether, the presented results are hypothesis generating and need to be verified in further trials.

## 5. Conclusions

The results of the present study suggest that the severity of alopecia areata may be associated with an increased risk of atherosclerosis. Further studies are necessary to evaluate atherosclerosis and cardiovascular risk in patients with alopecia areata.

## Figures and Tables

**Table 1:** Baseline patient characteristics and the assessment of cardiovascular and atherosclerosis markers.

Parameter	Patients with Alopecia Areata (*n* = 60)	Healthy Controls (*n* = 20)	Statistical Significance
**Cardiovascular and Atherosclerosis Markers**			
MPO (ng/mL), median (IQR)	91.27 (68.82–130.9)	78.95 (45.55–129.4)	0.21
IL1RL1 (pg/mL), median (IQR)	4628 (3886–5925)	4666 (3886–5193)	0.54
GDF15 (pg/mL), median (IQR)	40.84 (24.3–68.9)	37.35 (25.51–42.92)	0.40
CCL4 (pg/mL), median (IQR)	338. 3 (223.8–527.8)	285.2 (228.5–432.6)	0.19
CCL7 (pg/mL), median (IQR)	31.69 (20.12–48.13)	28.45 (21.52–36.59)	0.67
SORT1 (pg/mL), median (IQR)	297.8 (282.3–314.35)	312 (296.6–321.4)	0.09
**Demographic and Clinical Data**			
Age (years), mean ± SD	39 ± 15	37 ± 10	0.44
Sex (women), *n* (%)	41 (68%)	14 (70%)	0.88
BMI (kg/m^2^), median (IQR)	23.88 (21.09–26.88)	23.68 (19.46–25.37)	0.25
Smoking (yes), *n* (%)	10 (17%)	4 (20%)	0.73
Family history (yes), *n* (%)	12 (20%)	3 (15%)	0.61
Age at the first episode of alopecia (years), mean (range)	31 (3–70)	NA	
Number of episodes of hair loss, *n* (range)	3 (1–20)	NA	
Duration of the present episode of alopecia (months), mean (range)	30 (1–300)	NA	
SALT, mean (range)	43 (5–100)	NA	
Activity of the disease, n (%)		NA	
Active	25 (42%)		
Stable	27 (45%)		
Remitting	8 (13%)		
**Laboratory Parameters**			
Glucose (mg/dL), median (IQR)	88 (81–95.5)	84.5 (80–90)	0.26
Cholesterol (mg/dL), median (IQR)	186 (162–209)	190.5 (161–218.5)	0.55
LDL cholesterol (mg/dL), mean ± SD	120 ± 40	119 ± 39	0.96
HDL cholesterol (mg/dL), Median (IQR)	66.5 (55.5–81.5)	69.5 (57–83)	0.77
Triglycerides (mg/dL), median (IQR)	96 (66–143)	89 (58–139)	0.32

**Table 2:** Spearman’s correlation coefficients between selected clinical and laboratory parameters in patients with alopecia areata.

Parameter	Disease Duration (Months)	Age at Disease Onset (Years)	Number of Hair Loss Episodes (*n*)	SALT Score
MPO (ng/mL), median (IQR)	0.170	−0.132	0.955	0.214
IL1RL1 (pg/mL), median (IQR)	−0.130	0.787	−0.010	0.003
GDF15 (pg/mL), median (IQR)	−0.147	0.509 **	−0.016	−0.026
CCL4 (pg/mL), median (IQR)	0109	0.199	−0.067	−0.019
CCL7 (pg/mL), median (IQR)	0.048	0.174	0.011	0.281 *
SORT1 (pg/mL), median (IQR)	0.146	−0.099	0.162	0.013

* *p* = 0.03; ** *p* < 0.0001.

**Table 3:** Baseline characteristics and assessment of cardiovascular and atherosclerosis markers in patients with alopecia areata compared to healthy controls.

Parameter	Patients with SALT score < 50% (*n* = 35)	Healthy Controls (*n* = 20)	Statistical Significance
**Cardiovascular and Atherosclerosis Markers**			
MPO (ng/mL), median (IQR)	93.47 (78.77–136.8)	78.95 (45.55–129.4)	0.1
IL1RL1 (pg/mL), median (IQR)	4818 (3726–5341)	4666 (3886–5193)	0.75
GDF15 (pg/mL), median (IQR)	41.52 (21.62–65.28)	37.35 (25.51–42.92)	0.52
CCL4 (pg/mL), mean ± SD	382.49 ± 208.74	303.82 ± 176.08	0.17
CCL7 (pg/mL), median (IQR)	26.26 (18.20–43.82)	28.45 (21.52–36.59)	0.64
SORT1 (pg/mL), median (IQR)	297.8 (287.1–313.15)	312 (296.6–321.4)	0.15
**Demographic and Clinical Data**			
Age (years), mean ± SD	40 ± 15	37 ± 10	0.41
Sex (women), *n* (%)	22 (63%)	14 (70%)	0.59
BMI (kg/m^2^), median (IQR)	24.81 (21.77–27.77)	21.29 (19.46–25.37)	0.06
Smoking (yes), *n* (%)	6 (17%)	4 (20%)	0.79
Family history (yes), *n* (%)	8 (23%)	3 (15%)	0.48
**Laboratory Parameters**			
Glucose (mg/dL), median (IQR)	92 (83–101)	84.5 (80–90)	<0.05
Cholesterol (mg/dL), mean ± SD	191 ± 33	192 ± 34	0.91
LDL cholesterol (mg/dL), mean ± SD	124 ± 36	119 ± 30	0.64
HDL cholesterol (mg/dL), mean ± SD	64 ± 19	69 ± 19	0.27
Triglycerides (mg/dL), median (IQR)	96 (65–151)	89 (58–139)	0.94

**Table 4:** Baseline characteristics and assessment of cardiovascular and atherosclerosis markers in patients with a SALT score ≥ 50% compared to the healthy controls.

Parameter	Patients with SALT score ≥ 50% (*n* = 25)	Healthy Controls (*n* = 20)	Statistical Significance
**Cardiovascular and Atherosclerosis Markers**			
MPO (ng/mL), median (IQR)	82.78 (57.53–123.3)	78.95 (45.55–129.4)	0.48
IL1RL1 (pg/mL), median (IQR)	4513 (3966–6775)	4666 (3886–5193)	0.39
GDF15 (pg/mL), median (IQR)	37.57 (23.57–74.32)	37.35 (25.51–42.92)	0.67
CCL4 (pg/mL), mean ± SD	371.58 ± 181.81	303.82 ± 176.08	0.22
CCL7 (pg/mL), median (IQR)	38.91 (29.36–53.67)	28.45 (21.52–36.59)	0.11
SORT1 (pg/mL), median (IQR)	300.15 (281.1–314.35)	312 (296.6–321.4)	0.12
**Demographic and Clinical Data**			
Age (years), mean ± SD	39 ± 15	37 ± 10	0.58
Sex (women), *n* (%)	19 (76%)	14 (65%)	0.65
BMI (kg/m^2^), median (IQR)	22.46 (20.70–25.28)	21.29 (19.46–25.37)	0.45
Smoking (yes), *n* (%)	4 (16%)	4 (20%)	0.72
Family history (yes), *n* (%)	4 (16%)	3 (15%)	0.92
**Laboratory Parameters**			
Glucose (mg/dL), mean ± SD	87 ± 7	85 ± 9	0.55
Cholesterol (mg/dL), mean ± SD	189 ± 48	192 ± 34	0.78
LDL cholesterol (mg/dL), mean ± SD	113 ± 39	119 ± 30	0.57
HDL cholesterol (mg/dL), mean ± SD	70 ± 22	69 ± 19	0.97
Triglycerides (mg/dL), median (IQR)	95 (68–140)	89 (58–139)	0.34

**Table 5:** Selected clinical and laboratory parameters in patients with active, stable, and remitting alopecia areata.

Parameter	Progressive * (*n* = 25)	Stable ** (*n* = 27)	Remitting *** (*n* = 8)	Statistical Significance
**Atherosclerosis and Cardiovascular Risk Markers**				
MPO (ng/mL), median (IQR)	85.21 (65.74–112.7)	91.69 (73.53–137.10)	101.62 (65.85–136.20)	0.77
IL1RL1 (pg/mL), median (IQR)	4969 (3966–5997)	4474.5 (3966–5193)	3645 (3400–7466)	0.66
GDF15 (pg/mL), median (IQR)	47.08 (26.48–69.36)	31.71 (20.14–63.48)	61.64 (24.54–82.44)	0.3
CCL4 (pg/mL), mean ± SD	360.46 ± 210.75	388.23 ± 199.43	394.48 ± 157.15	0.86
CCL7 (pg/mL), median (IQR)	29.64 (18.84–45.92)	31.96 (18.84–48.25)	34.5 (28.39–50.79)	0.61
SORT1 (pg/mL), median (IQR)	302.5 (285.9–316.7)	297.8 (281.1–312)	293 (276.3–319)	0.58
**Demographic and Clinical Data**				
Age (years), mean ± SD	37 ± 16	43 ± 15	36 ± 9	0.28
Sex (women), *n* (%)	13 (52%)	22 (81%)	6 (75%)	0.06
BMI (kg/m^2^), median (IQR)	24.37 (21.96–27.68)	23.73 (20.70–29.76)	21.77 (20.95–24.63)	0.30
Smoking (yes), *n* (%)	5 (20%)	4 (15%)	1 (13%)	0.83
Family history (yes), *n* (%)	6 (24%)	5 (19%)	1 (13%)	0.75
**Laboratory Data**				
Glucose (mg/dL), median (IQR)	92 (85–101)	84 (79–94)	86.5 (85–102)	0.04
Cholesterol, mean ± SD	186 ± 29	192 ± 52	196 ± 22	0.81
LDL cholesterol (mg/dL), mean ± SD	122 ± 30	119 ± 46	116 ± 28	0.92
HDL cholesterol (mg/dL), median (IQR)	60 (49–73)	63 (51–81)	80 (76–84.5)	0.04
Triglycerides (mg/dL), median (IQR)	97 (68–151)	97 (65–143)	71.5 (57–138)	0.62

* an increase in total hair loss of more than 5% over the month prior to the examination; ** a change in total hair loss of less than 5% over the month prior to the examination; *** a decrease in total hair loss of more than 5% over the month prior to the examination.

## Data Availability

Not applicable.
